# Surgeon experience and patient selection influence the learning curve in robotic inguinal hernia repair

**DOI:** 10.1007/s11701-025-02910-4

**Published:** 2025-10-30

**Authors:** Victor Rodrigues-Gonçalves, Mireia Verdaguer-Tremolosa, Pilar Martínez-López, Manuel López-Cano

**Affiliations:** General Surgery Department, Abdominal Wall Surgery Unit, Hospital Universitari Vall d´Hebron, Universitat Autònoma de Barcelona, Paseo Vall d`Hebron 119-129, Barcelona, 08035 Spain

**Keywords:** Robotic-assisted surgery, Inguinal hernia repair, Learning curve, Surgical training, Operative time

## Abstract

Robotic-assisted laparoscopic inguinal hernia repair offers ergonomic and technical advantages over conventional approaches, but its adoption requires a learning process influenced by multiple factors. While prior surgical experience is a known determinant of performance, the impact of patient selection and hernia complexity on the learning trajectory remains insufficiently explored. This retrospective single-center study evaluated the learning curves of three surgeons with varying levels of prior open, laparoscopic, and robotic experience, who performed robotic-assisted transabdominal preperitoneal (rTAPP) inguinal hernia repairs between September 2018 and May 2023. Operative time was plotted against sequential case number to identify distinct phases in each surgeon’s learning curve. Associations between clinical variables and curve phases were analyzed statistically. Additional descriptive analyses were performed for unilateral and bilateral hernias. A total of 110 consecutive patients were included. Surgeons A and B (more experienced) demonstrated two-phase learning curves, while Surgeon C (less experienced) exhibited a four-phase curve with greater variability in operative time. Differences in patient and hernia complexity across phases were most notable in Surgeon C’s cases. The learning curve for rTAPP is modulated not only by prior surgical experience, but also by the evolving complexity of patient selection during training. These findings support the implementation of structured training programs that prioritize progressive case selection, beginning with less complex hernias, to ensure safe and efficient skill acquisition.

## Introduction

Inguinal hernia repair remains one of the most common surgical procedures worldwide [1]. While laparoscopic techniques have demonstrated benefits in terms of postoperative recovery and reduced pain, their steep learning curve and technical limitations have hindered widespread adoption [2]. Robotic-assisted surgery offers improved visualization, enhanced ergonomics, and greater instrumental precision—advantages that may be particularly beneficial in complex inguinal hernias [3].

As with any advanced surgical technique, robotic-assisted laparoscopic inguinal hernia repair involves a learning curve. This is typically defined as the progression through distinct performance phases, beginning with an initial period of skill acquisition and culminating in technical proficiency, often measured through operative time, complication rates, or other performance indicators [4]. The structure and duration of the learning curve can vary substantially and are influenced by multiple factors, including prior surgical experience (open, laparoscopic, or robotic), frequency of case exposure, case complexity, institutional support, and the presence of structured training programs [5].

Importantly, surgeon experience alone may not fully explain variations in operative performance. Patient-related factors (such as bilateral or inguinoscrotal hernias, history of abdominal surgery, or significant comorbidities) can introduce technical challenges that impact operative efficiency, especially in early stages of learning [6]. These elements are rarely standardized in studies of learning curves but may critically influence surgical outcomes and perceived proficiency.

While prior work in laparoscopic hernia repair (TEP technique) has highlighted the importance of patient selection in early training phases [7], this aspect has been insufficiently explored in the context of robotic-assisted repair. Most published studies on robotic-assisted transabdominal preperitoneal hernia repair (rTAPP) have focused on case volume or prior laparoscopic experience as determinants of proficiency, with limited attention to how surgeons choose patients during the learning process [8].

The aim of this study is to compare the learning curves of robotic-assisted laparoscopic inguinal hernia repair among surgeons with different levels of prior surgical experience and to explore how decisions regarding patient and hernia selection influence the evolution of operative performance.

## Methods

This is a retrospective single-center study conducted at the Abdominal Wall Surgery Unit of a high-volume academic center. The Abdominal Wall Surgery Unit has been accredited [8] and has had a dedicated robotic surgery program since September 2018. The implementation of this program was carried out in line with the training pathway of the European Hernia Society (EHS) for initiating robotic-assisted abdominal wall surgery programs [9].

The study was designed in accordance with the Reporting of Studies Conducted Using Observational Routinely-Collected Data (RECORD) [10] and Strengthening the Reporting of Observational Studies in Epidemiology (STROBE) [11] statements, ensuring compliance with current methodological standards for observational research. The study protocol was reviewed and approved by the Institutional Review Board and Ethics Committee (code number PR [AG] 392/2025).

### Patient selection

All consecutive adult patients (≥ 18 years) who underwent elective rTAPP inguinal hernia repair between September 2018 and May 2023 were identified from the prospectively maintained clinical database of the unit and considered for inclusion. Both unilateral and bilateral repairs were eligible. Patients were excluded if operative time was not recorded or if relevant clinical data were incomplete. All included patients were preoperatively assessed and deemed fit for elective surgery according to institutional protocols. The decision to include a patient during the learning curve period was made independently by each surgeon.

### Data collected

Demographic, clinical, surgical, and postoperative variables were collected retrospectively from the hospital’s electronic medical records and entered into a structured research database. Demographic variables included age, sex, and body mass index (BMI). Clinical data comprised the American Society of Anesthesiologists (ASA) classification, comorbidities (cardiovascular, renal, and respiratory diseases), use of anticoagulants, and history of previous abdominal surgery.

Hernia-related variables included the type of hernia (classified as lateral, medial, or femoral); the side involved (right or left); and whether the presentation was unilateral or bilateral. Additionally, we recorded whether the hernia was inguinoscrotal and whether it was classified as recurrent. A recurrent hernia was defined as a new herniation at the same anatomical site following a previous surgical repair, regardless of the original technique. In this series, all recurrences occurred after previous open anterior mesh repair.

The primary outcome was operative time, defined as the interval between skin incision and skin closure (“skin-to-skin”), which was used as an objective surrogate for surgical proficiency. Secondary outcomes included postoperative complications (specifically global complications, hematoma, and seroma), which were analyzed descriptively due to their low frequency.

Operative data also included the surgeon performing the procedure and the sequential case number for each surgeon reflecting cumulative experience.

### Surgeons and surgical technique

All procedures were performed by three surgeons, each with different levels of surgical experience. Surgeon A was a senior consultant with extensive experience in open and minimally invasive abdominal wall surgery. Surgeon B was a mid-career consultant with moderate experience in open and minimally invasive surgery. Surgeon C was an early-career surgeon with limited prior surgical experience in open and minimally invasive surgery. All of them had the theoretical and practical knowledge necessary to use the robotic platform. Each surgeon performed the procedures independently during the study period and their individual learning curves were analyzed separately.

The robotic platform used was either Da Vinci Xi or Da Vinci X depending on availability during surgical scheduling (Intuitive Surgical, Inc., Sunnyvale, CA). All surgeries were performed using a rTAPP approach, following the standard steps described for laparoscopic inguinal hernia repair [12]. The procedure was carried out under general anesthesia, with the patient positioned in a supine Trendelenburg position. A three-port technique was used, with the robotic ports placed according to the typical TAPP configuration. After entering the peritoneal cavity, the hernia sac was carefully dissected in all cases and the myopectineal orifice was completely exposed.

A flat non-absorbable synthetic mesh with wide pores and medium weight (15 × 12 cm) was placed to cover the defect and the myopectineal region. Mesh fixation was performed in most cases using a single stitch of slowly absorbable suture material anchored to the pectineal (Cooper’s) ligament. The peritoneum was then closed with running barbed sutures. The same standardized technique was applied in all cases, regardless of the surgeon’s level of experience. For bilateral hernia repairs, two separate peritoneal flaps were created (one for each side) and each hernia was addressed independently, following the same surgical principles described above.

### Learning curve analysis

Learning curves were generated for each surgeon by plotting operative time (in minutes) against the chronological case sequence. The curves were examined and segmented into distinct phases defined by increasing or decreasing trends in operative time, while periods of stability were excluded from segmentation. This methodological approach enabled the assessment of performance dynamics, potentially reflecting skill acquisition, technical challenges associated with specific cases, or variations in case complexity over time.

Three complementary sets of curves were generated for each surgeon. The first included all cases (both unilateral and bilateral hernias) and was used to identify learning phases and conduct formal statistical comparisons of clinical variables across phases. The second and third curves displayed only unilateral and bilateral hernia repairs, respectively, and served for descriptive and visual analysis of operative time trends in procedures of differing complexity. Due to sample size limitations in these subgroups, no statistical comparisons were conducted for the unilateral and bilateral curves.

### Statistical analysis

Descriptive statistics were used to summarize the study population. Continuous variables were expressed as mean and standard deviation (SD), while categorical variables were presented as absolute frequencies and percentages. Comparisons between different learning curve phases were performed using the chi-square or Fisher’s exact test for categorical variables, and the Mann–Whitney U or Kruskal–Wallis test for continuous variables, depending on the number of groups. A two-sided p-value < 0.05 was considered statistically significant. Learning curves were constructed by plotting operative time (in minutes) against the sequential case number for each surgeon. Curves were visually segmented into distinct phases based on ascending or descending trends in operative time, and comparisons between phases were made to explore associations with clinical variables. All analyses were performed using IBM SPSS Statistics 23.

## Results

### Patient characteristics

Between September 2018 and May 2023, a total of 110 patients underwent robotic-assisted laparoscopic inguinal hernia repair and were included in the study. The mean age was 69.6 years (SD 13.5), and 82% (*n* = 90) were male. The mean BMI was 25.9 kg/m² (SD 3.0). Regarding ASA classification, 67% (*n* = 74) of patients were classified as ASA I/II and 33% (*n* = 36) as ASA III/IV. Comorbidities were common: 67% (*n* = 74) of patients had cardiovascular disease, 18% (*n* = 20) had diabetes mellitus, 12% (*n* = 13) had chronic kidney disease, and 11% (*n* = 12) had chronic obstructive pulmonary disease. Additionally, 18% (*n* = 20) of patients were on anticoagulant therapy, and 29% (*n* = 32) had a history of previous abdominal surgery.

Concerning hernia characteristics, lateral hernias represented 65% (*n* = 72) of cases, medial hernias 29% (*n* = 32), and femoral hernias 6% (*n* = 6). Bilateral hernia repair was performed in 36% (*n* = 40) of patients, 26% (*n* = 29) had inguinoscrotal hernias, and 10% (*n* = 11) presented with recurrent hernias. All recurrent hernias included in this study occurred after prior open mesh repairs; no patients had previously undergone laparoscopic or robotic hernia repair.

Patient distributions varied across the three surgeons (Table 1). Surgeon A treated a group with lower variability in ASA classification and comorbidity profile. Surgeon B operated on a higher proportion of patients with ASA III/IV and cardiovascular disease. Surgeon C’s group included a wider range of patient characteristics, including higher variability in ASA scores, comorbidities, and previous abdominal surgeries.

**Table 1:** Patient characteristics of study population

Variables	Total (*n*= 110)	Surgeon A group (*n*= 20)		Surgeon B group (*n*= 57)	Surgeon C group (*n*= 33)	*P* values
**Age (yr)[mean (SD)]**	69.6 (13.53)	71.45 (13.59)	71.32 (11.12)		65.58 (16.52)	0.122
**Sex [n**,** (%)]** **Male** **Female**	90 (82) 20 (18)	14 (70) 6 (30)	49 (86) 8 (14)		27 (82) 6 (18)	0.281
**BMI (kg/m** ^**2**^ **) [mean (SD)]**	25.86 (3.02)	25.65 (3.04)	25.49 (2.74)		26.64 (3.41)	0.209
**ASA score** **I/II [n**,** (%)]** **III/IV [n**,** (%)]**	74 (67) 36 (33)	16 (80) 4 (20)	32 (56) 25 (44)		26 (79) 7 (21)	0.040
**Previous abdominal surgery [n**,** (%)]**	32 (29)	4 (20)	16 (28)		12 (36)	0.423
**Comorbidity [n**,** (%)]**	83 (76)	13 (65)	48 (84)		22 (67)	0.086
**Cardiovascular disease [n**,** (%)]**	74 (67)	11 (55)	45 (79)		18 (55)	0.026
**Chronic obstructive pulmonary disease [n**,** (%)]**	12 (11)	1 (5)	7 (12)		4 (12)	0.778
**Chronic nephropathy [n**,** (%)]**	13 (12)	1 (5)	11 (19)		1 (3)	0.048
**Liver cirrhosis [n**,** (%)]**	3 (3)	1 (5)	2 (4)		0 (0)	0.582
**Diabetes [n**,** (%)]**	20 (18)	2 (10)	10 (18)		8 (24)	0.456
**Active smoking [n**,** (%)]**	14 (13)	1 (5)	6 (11)		7 (21)	0.229
**Anticoagulant treatment [n**,** (%)]**	20 (18)	0 (0)	15 (26)		5 (15)	0.017
**Comorbidity more than one [n**,** (%)]**	47 (43)	4 (20)	32 (56)		11 (33)	0.008
**Hernia type [n**,** (%)]** **Lateral** **Medial** **Femoral**	71 (65) 32 (29) 7 (6)	14 (70) 4 (20) 2 (10)	36 (63) 18 (32) 3 (5)		21 (64) 10 (30) 2 (6)	0.833
**Bilateral hernia repair [n**,** (%)]**	39 (36)	10 (50)	21 (37)		8 (24)	0.156
**Unilateral hernia repair [n**,** (%)]** **Right** **Left**	42 (38) 30 (27)	4 (20) 6 (30)	22 (39) 15 (26)		16 (49) 9 (27)	0.267
**Inguinoescrotal hernia [n**,** (%)]**	28 (26)	7 (35)	11 (19)		10 (30)	0.285
**Recurrent hernia [n**,** (%)]**	11 (10)	2 (10)	6 (11)		3 (9)	1.000
**Operative time (min) [mean (SD)]**	135.1 (42.1)	148.8 (49.5)	131.1 (40.6)		133.64 (39.3)	0.264
**Postoperative complication [n (%)]**	31 (28)	7 (35)	14 (25)		10 (30)	0.637
**Hematoma [n**,** (%)]**	6 (6)	1 (5)	5 (9)		0 (0)	0.209
**Seroma [n**,** (%)]**	25 (23)	7 (35)	8 (14)		10 (30)	0.073
**Length of stay (days) [mean (SD)]**	1 (0.2)	1 (0.1)	1 (0.2)		1 (0.3)	0.632

Postoperative complications were recorded and are presented in Table 1. A total of 31 patients (28%) experienced any complication, most commonly seroma (23%) or hematoma (6%). Due to low event rates, no formal statistical comparisons were conducted between learning curve phases.

### Learning curve analysis

**Fig. 1:**
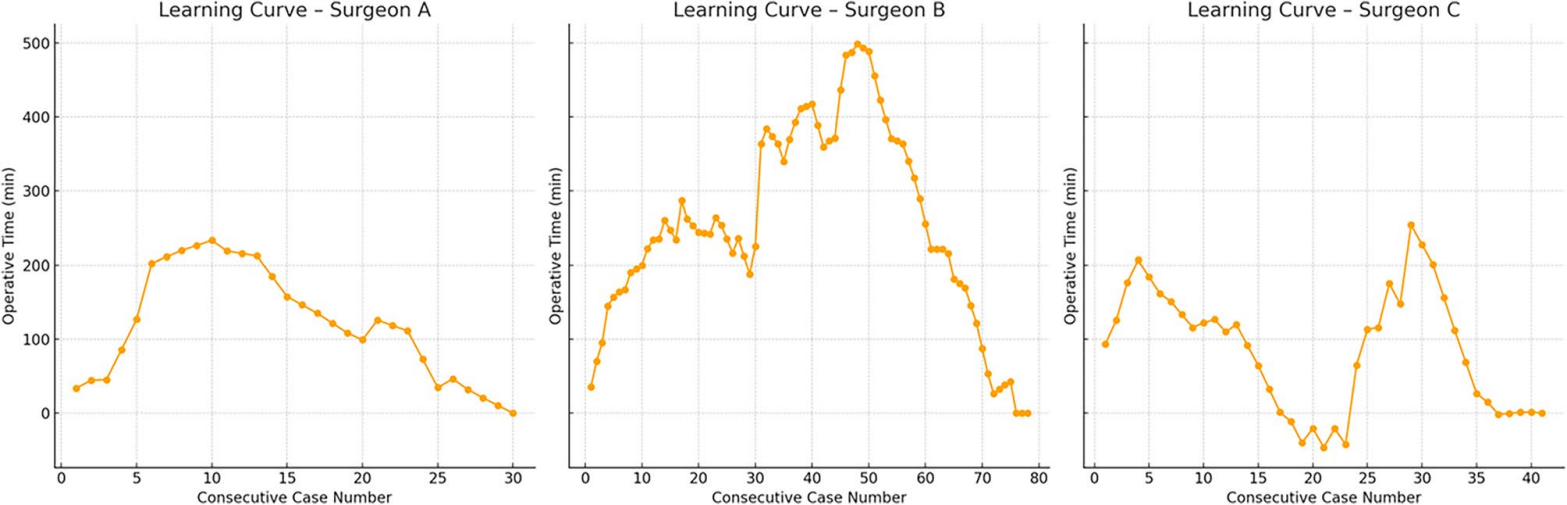
Learning curves for robotic-assisted repair of all inguinal hernias: surgeons A, B, and C

Three sets of learning curves were generated for each surgeon. The first set included all cases (unilateral and bilateral) and was segmented into distinct phases based on increases or decreases in operative time. These were used for formal comparisons between phases. Surgeon A had two phases (11 and 29 cases), Surgeon B also had two (15 and 25 cases), and Surgeon C presented a more complex curve with four phases (6, 11, 8, and 10 cases). Learning curves declined for all surgeons but trajectories differed with respect to case selection. Surgeon A, who operated on a relatively homogeneous case mix showed a rapid reduction in operative time with visual stabilization and narrow late-phase dispersion. Surgeon B also treated a fairly homogeneous cohort but exhibited a prolonged learning phase with marked mid-series variability but cases operative times decreased consistently and stabilized with low variance indicating a robust proficiency point. In contrast, Surgeon C managed a more heterogeneous case mix, with alternating clusters of easier and more complex procedures. Although the curve suggests an apparent early plateau subsequent surges in operative time coincide with more complex cases, implying that this early “proficiency” is likely confounded by case-selection heterogeneity rather than consolidated skill. Overall, while C appears to reach proficiency earlier, B’s later proficiency is more stable and potentially more generalizable across varying case complexity, and A achieves an early, steady-state pattern under a more uniform case profile. (Fig. 1).

**Fig. 2:**
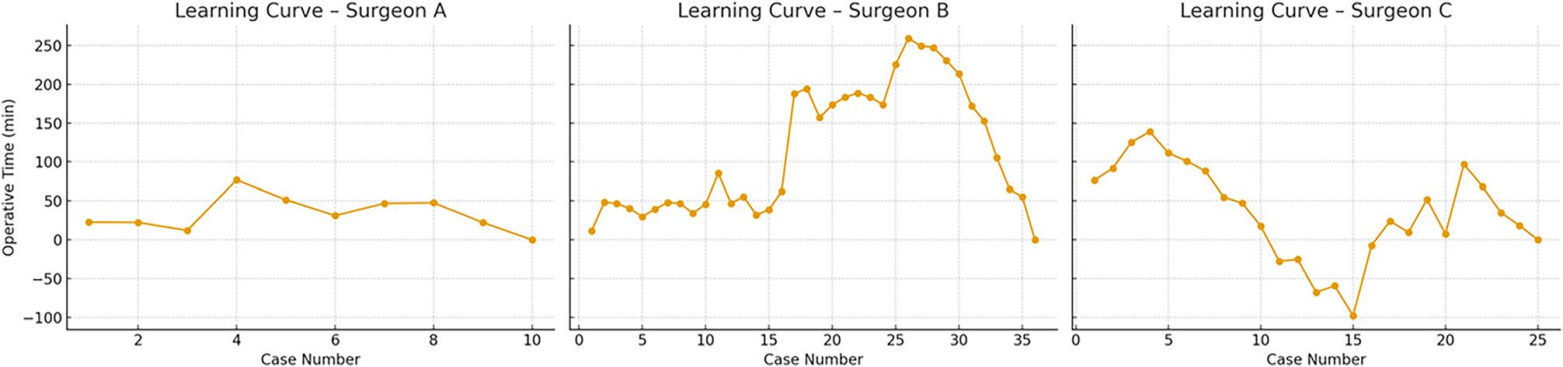
Learning curves for robotic repair of unilateral inguinal hernias by surgeons A, B, and C

**Fig. 3:**
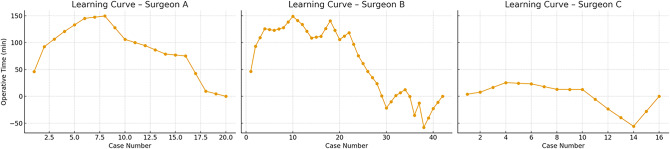
Learning curves for bilateral inguinal hernias repair by surgeons A, B, and C

Additionally, separate learning curves were plotted for unilateral and bilateral hernia repairs for each surgeon (Figs. 2 and 3). These curves were used for descriptive and visual interpretation only, not for statistical comparisons. Across all surgeons, unilateral cases showed more stable and shorter operative times, whereas bilateral cases exhibited greater variability, especially in the case of Surgeon C.

### Association between learning curve phases and clinical factors

The distribution of clinical and hernia-related variables across learning curve phases differed among surgeons (Table 2). For Surgeon A, no statistically significant differences were found between phases in any of the analyzed variables, which is consistent with the stable trajectory observed in their learning curve (Fig. 1).

**Table 2:** Clinical and hernia-related characteristics of patients across the learning curve phases for each surgeon (global curve analysis)

Variables	Surgeon A group (*n*= 30)		*P* values	Surgeon B group (*n*= 78)		*P* values	Surgeon C group (*n*= 41)				*P* values
	Phase 1 (*n*= 10)	Phase 2 (*n*= 20)		Phase 1	Phase 2		Phase 1	Phase 2	Phase 3	Phase 4	
**Age (yr)[mean (SD)]**	69.3 (10.1)	70.9 (15.9)	0.782	70.8 (11.7)	70.7 (10.9)	0.985	68.5 (12.2)	59.4 (18.5)	72.3 (6.7)	68.2 (13.9)	0.215
**Sex [n**,** (%)]** **Male** **Female**	9 (10) 1 (10)	13 (65) 7 (35)	0.210	44 (92) 4 (7)	23 (77) 7 (23)	0.094	4 (100) 0 (0)	15 (79) 4 (21)	7 (100) 0 (0)	7 (64) 4 (36)	0.268
**BMI (kg/m** ^**2**^ **) [mean (SD)]**	25.9 (2.5)	25.8 (3.1)	0.853	25.9	25.4 (2.8)	0.449	28.1 (1.9)	26.8 (3.8)	27.4 (3.8)	24.9 (2.5)	0.314
**ASA score** **I/II [n**,** (%)]** **III/IV [n**,** (%)]**	8 (80) 2 (20)	16 (80) 4 (20)	1.000	29 (60) 19 (40)	19 (63) 11 (37)	0.816	3 (79) 1 (25)	15 (79) 4 (21)	5 (71) 2 (29)	10 (91) 1 (9)	0.703
**Previous abdominal surgery [n**,** (%)]**	0 (0)	6 (30)	0.074	12 (25)	11 (34)	0.313	1 (25)	4 (21)	2 (29)	9 (82)	0.007
**Comorbidity [n**,** (%)]**	8 (80)	11 (55)	0.246	44 (85)	21 (70)	0.149	4 (100)	11 (58)	4 (57)	9 (82)	0.306
**Cardiovascular disease [n**,** (%)]**	8 (80)	9 (45)	0.119	38 (79)	19 (63)	0.189	4 (100)	9 (47)	4 (57)	4 (36)	0.210
**Anticoagulant treatment [n**,** (%)]**	0 (0)	0 (0)	-	12 (25)	4 (13)	0.260	0 (0)	1 (5)	2 (29)	2 (18)	0.305
**Hernia type [n**,** (%)]** **Lateral** **Medial** **Femoral**	8 (80) 2 (20) 0 (0)	15 (75) 3 (15) 2 (10)	1.000	30 (63) 15 (31) 3 (6)	18 (60) 12 (40) 0 (0)	0.382	3 (75) 1 (25) 0 (0)	10 (53) 8 (42) 1 (5)	5 (71) 2 (29) 0 (0)	5 (46) 4 (36) 2 (18)	0.828
**Bilateral hernia repair [n**,** (%)]**	6 (60)	14 (70)	0.690	20 (42)	22 (73)	0.010	0 (0)	2 (11)	5 (71)	4 (36)	0.009
**Inguinoescrotal hernia [n**,** (%)]**	5 (50)	7 (35)	0.461	10 (21)	4 (13)	0548	0 (0)	8 (42)	1 (14)	7 (64)	0.085
**Recurrent hernia [n**,** (%)]**	0 (0)	3 (15)	0.532	5 (10)	4 (13)	0.727	0 (0)	2 (11)	1 (14)	1 (9)	1.000

In contrast, Surgeon B showed a significantly higher proportion of bilateral hernias in the second phase compared to the first (73% vs. 42%, *P* = 0.010), coinciding with a slight increase in operative time in the latter part of the curve (Fig. 1).

Surgeon C displayed the most complex learning pattern, with four distinct phases and multiple changes in operative time (Fig. 1). Several clinical factors varied significantly across phases, particularly the proportion of bilateral hernias (*P* = 0.009) and previous abdominal surgeries (*P* = 0.007), both of which were more common in the later phases. Interestingly, phase 4 showed a decrease in operative time despite a higher frequency of complex cases.

## Discussion

Prior evidence in laparoscopic TEP repair indicates that surgeons in the early learning phase preferentially select “favorable” cases (younger, slender patients with unilateral, non-scrotal primary hernias and no prior abdominal surgery) to reduce operative difficulty, conversions, and complications. BMI has been identified as a difficulty modifier specifically during the learning period, supporting its use as a pragmatic selection criterion at the outset [6]. Although robotic rTAPP learning curves have been described and appear influenced by prior laparoscopic experience and team efficiency [5, 7, 13, 14], explicit analyses linking surgeon experience to initial patient selection remain limited, highlighting an area for further study [6, 7, 15].

Three key findings emerged from our study. First, surgeons with greater prior experience in open and laparoscopic surgery demonstrated smoother and more stable learning curves, with fewer fluctuations in operative time, probably associated with a more uniform patient profile. Second, less experienced surgeons showed more complex patterns, with multiple inflection points and greater variability, likely due to more heterogeneous case selection. Third, clinical and hernia-related factors (particularly bilateral hernias and a history of previous abdominal surgery) were significantly associated with specific phases of the learning curves.

Previous studies on robotic inguinal hernia repair have primarily focused on the number of cases required to reach proficiency, commonly using operative time as the primary indicator of performance. Reported thresholds vary widely, ranging from as few as 20 to over 60 cases, largely depending on the surgeon’s baseline experience and case complexity [5, 7, 16]. Our findings align with this variability: the most experienced surgeons demonstrated stabilization after approximately 10–15 cases, while the less experienced surgeon showed a longer and more irregular trajectory. Similar patterns have been described in laparoscopic hernia repair and robotic colorectal surgery, where operative performance is influenced not only by technical familiarity but also by patient complexity and procedural demands [17]. These observations support the notion that learning curves in minimally invasive surgery are dynamic and context-dependent and that extrapolating case numbers across procedures may be misleading.

Several factors have been identified in the literature as influencing the learning curve in rTAPP, including prior laparoscopic experience, familiarity with robotic platforms, standardized procedural steps, and institutional case volume [13, 14]. Technical aspects such as docking time, console coordination, and ergonomics can also affect the pace and stability of skill acquisition, particularly during the early learning phase. Although direct evidence in robotic inguinal hernia repair is limited, simulation-based training and preclinical exposure have been reported in other robotic surgical fields to enhance coordination and technical performance, suggesting their potential role in facilitating the transition to clinical practice [18]. However, further studies are needed to confirm the specific impact of simulation and preclinical training on rTAPP learning outcomes [19].

The present study also underscores the role of clinical and hernia-related factors (i.e., patient selection) in shaping the learning trajectory. Among these, bilateral hernias and previous abdominal surgeries were most consistently associated with changes in operative time across phases, particularly for less experienced surgeons with more variable selection criteria. These findings suggest that such complex cases are best introduced in the later stages of the learning curve, once a stable level of technical proficiency has been attained. This approach may help prevent unnecessary prolongation of operative time and reduce potential risks. Similar observations have been made in laparoscopic hernia repair where bilateral procedures and prior abdominal surgeries are known to increase technical demands and operative duration [20, 21]. Additionally, it is important to recognize that surgical performance is influenced not only by the surgeon, but also by parallel learning processes among the operating room staff, including anesthesiologists and nurses. While these factors were not formally assessed in this study, their contribution to operative efficiency should not be overlooked [22].

From a training perspective, our results support the concept that rTAPP for simple unilateral hernias represents the optimal starting point for robotic hernia programs, as previously suggested in the literature [9], and that the surgeon’s experience in selecting these cases is essential. This standardized and relatively short procedure enables the development of key robotic skills, including docking, dissection, mesh handling, and suturing. More complex cases, such as bilateral or inguinoscrotal hernias, should be gradually incorporated once surgeons demonstrate technical stability. This staged approach aligns with structured training models that promote progressive case allocation and even suggest dividing rTAPP into discrete procedural steps for objective assessment [23]. Importantly, the variability in reported thresholds for achieving proficiency (ranging from 20 to more than 60 cases) highlights the limitations of fixed numerical benchmarks. Instead, surgical training should emphasize learning based on an appropriate and homogeneous selection of patients that allows individualized progression, competency-based evaluation, and continuous monitoring of learning curves to ensure safe and effective adoption of robotic techniques.

This study has several limitations. First, the retrospective and single-center design inherently limits the generalizability of the findings. Second, operative time was used as the primary surrogate of proficiency; while practical and objective, it does not capture all dimensions of surgical performance such as precision, complication rates, or functional outcomes. Although postoperative complications were recorded, their low frequency precluded meaningful statistical analysis, and other relevant outcomes (such as recurrence or patient-reported measures) were not available in a standardized format. Third, the modest sample size constrained the use of multivariable models and required grouping unilateral and bilateral cases for certain statistical comparisons. Additionally, while the study included key preoperative variables such as ASA class, comorbidities, and prior surgeries, other potential confounders—particularly intraoperative factors like docking time, console time, or team experience—were not systematically recorded and could not be included in the analysis. Fourth, the study did not employ cumulative sum (CUSUM) analysis, a frequently used method for detecting inflection points in learning curves. However, our aim was to examine broader trends in operative performance and case selection patterns, which are not always adequately captured by single-threshold statistical models such as CUSUM. Finally, we acknowledge the absence of detailed information on prior robotic simulation training or hands-on exposure to robotic cases before the study period. Similarly, the influence of non-surgeon factors—such as anesthetic protocols, nursing staff experience, and operating room logistics—could not be quantified, although they likely contributed to the observed variability in operative times.

Despite these limitations, this study contributes to the scarce body of evidence on learning curves in robotic inguinal hernia repair. A notable strength is the direct comparison of surgeons with varying levels of prior surgical and robotic experience, which offers valuable insight into how baseline expertise shapes the learning process. Furthermore, the integration of patient and hernia-related factors provides a more nuanced understanding of how case complexity influences surgical performance.

In conclusion, robotic-assisted inguinal hernia repair requires a structured and progressive learning process, where both surgeon experience and thoughtful patient selection play central roles. Our findings indicate that surgeons with greater baseline experience tend to achieve smoother and more efficient learning curves. Starting with simple cases and advancing gradually to more complex scenarios (through a stepwise and homogeneous approach) may support safer skill acquisition and optimize operative performance. These insights may help guide future research aimed at refining patient selection criteria and improving training pathways for surgeons adopting rTAPP.

## Data Availability

No datasets were generated or analysed during the current study.
